# Assessment of help value affects reciprocation in Norway rats

**DOI:** 10.1098/rsos.231253

**Published:** 2023-10-25

**Authors:** Sacha C. Enghelhardt, Niklas I. Paulsson, Michael Taborsky

**Affiliations:** ^1^ Behavioural Ecology, Institute of Ecology and Evolution, University of Bern, Wohlenstrasse 50a, 3032 Hinterkappelen, Switzerland; ^2^ Department of Sociobiology/Anthropology, Johann-Friedrich-Blumenbach Institute for Zoology and Anthropology, University of Göttingen, Kellnerweg 6, 37077 Göttingen, Germany; ^3^ Behavioural Ecology and Sociobiology Unit, German Primate Center, Leibniz Institute for Primate Research, 37077 Göttingen, Germany; ^4^ Department of Collective Behavior, Max Planck Institute of Animal Behavior, 78467 Konstanz, Germany; ^5^ Institute for Advanced Study (Wissenschaftskolleg zu Berlin), 14193 Berlin, Germany

**Keywords:** cooperation, direct reciprocity, food provisioning, helping, quality–quantity trade-off, trading

## Abstract

Wild-type Norway rats reciprocate help received in a well-replicated experimental food-giving task, but the criteria to appraise the received help's value are unclear. We tested whether quality or quantity of received help is more important when deciding to return help, and whether partner familiarity and own current need affect this evaluation. We experimentally varied recipients of help's hunger state, and familiar or unfamiliar partners provided either higher caloric food (enhanced quantity; carrots) or food higher in protein and fat (enhanced quality; cheese). Reciprocation of received help was our criterion for the rats' value assessment. Familiarity, food type and hunger state interacted and affected help returned by rats. Rats returned less help to familiar partners than to unfamiliar partners. With unfamiliar partners, rats returned more help to partners that had donated preferred food (cheese) than to partners that had donated less preferred food (carrots), and they returned help earlier if they were satiated and had received cheese. With familiar partners, food-deprived rats that had received cheese returned more help than satiated rats that had received carrots. Our results suggest that Norway rats assess the received help's value based on its quality, their current need and partner familiarity before reciprocating received help.

## 1. Introduction

Norway rats are known to reciprocate help they received to obtain food in an iterated Prisoner's Dilemma paradigm [1–10], and they were shown to trade different commodities with each other [11]. Reciprocal trading in animals is affected by the quality and quantity of commodities [12–16]. In addition, other factors like current demands and the familiarity of social partners should take effect when deciding to return received goods and services [17–20]. Currently, the parameters influencing the decision of animals to return received help are not clear. Norway rats serve as an ideal model to promote our understanding of how cooperative decisions are made in an interative interaction scenario because of their social nature, high cooperation propensity and experimental accessibility.

Stable levels of cooperation can be established if partners apply the direct reciprocity decision rule, [21–23], and wild-type Norway rats (*Rattus norvegicus*) were shown to give more, and earlier, help to previously cooperative partners than to previously uncooperative partners [1,6,24]. In addition, they adjust their help both to the quality of help previously received [25] and to their partner's need for help [2,18]. This raises two questions: (i) how do receivers of help determine the value of received help and (ii) is the perceived value of received help dependent on the current need of the receiver? We addressed these questions by measuring help given by focal rats to partners in a sequential iterated Prisoner's Dilemma paradigm [26,27].

Due to the plethora of contentious terms in the study of cooperation and the abundance of divergent connotations, we deem it proper to provide a brief glossary of our use of relevant terms in this paper. *Altruism* is defined by the immediate consequences of an action as a behaviour or trait by which an individual (actor) benefits someone else (receiver(s)) at some immediate cost to itself [22,23]. This does not make assumptions about whether and how these costs may be compensated by e.g. future benefits (i.e. reciprocal altruism) or fitness benefits to non-descendant relatives (indirect fitness effects) [22,23]. *Cooperation* is the simultaneous or consecutive acting of two or more individuals by the same or different behaviours to achieve a (shared) goal [22,23]. Costs and benefits to either partner are not implied, i.e. the net fitness benefits of cooperation may or may not accrue to one, several or all involved parties [22,23]. *Reciprocity* is synonymous with *reciprocal cooperation*. This is essentially a proximate (i.e. mechanistic) concept implying decision rules evolved through certain cost/benefit relationships [22,23]. At the ultimate (i.e. evolutionary) level, this term refers to an apparently cooperative trait or behaviour that benefits a receiver of an act at immediate costs to the actor [22,23]. At the same time, it increases the probability of receiving benefits in return, from the same or different partners [22,23]. Reciprocation is hence intrinsically altruistic and prone to cheating [22,23]. At the proximate level, there are three forms of reciprocity implying different decision rules [22,23]: (i) generalized reciprocity denotes that individuals help anyone after receiving help from someone [28–32], (ii) direct reciprocity denotes that individuals help someone who has previously helped them [26,33] and (iii) indirect reciprocity denotes that individuals help someone who is helpful, implying a contingency on a reputation to be cooperative [30,34,35]. *Help* is an action by an individual to the apparent benefit of one or several receivers, and this term is devoid of assumptions about potential costs to the actor [22,23]. If cooperative actions are performed in sequence, i.e. mutual help is provided between social partners involving a time delay between subsequent interactions, cheating is a profitable temptation [22,36,37]. This situation is appropriately modelled by the *sequential iterated Prisoner's Dilemma game* [26,27], which is often used to study the decision rules of reciprocity [1,11,25,38]. Experiments assessing the reciprocal trading of different commodities are scarce [11,25,39]. Whether the quality or the quantity of help received and their interaction with the recipient's need affect help given is currently unknown.

Several studies have found that female Norway rats help unfamiliar partners according to the direct and generalized reciprocity decision rules [1–4,6,7,9,11,24,40,41], whereas male Norway rats apply the direct reciprocity decision rule, but not the generalized reciprocity decision rule [5,8]. In experiments testing for prosocial behaviour of rats without the possibility to reciprocate received help [42], laboratory strains of Norway rats helped partners enclosed in a tube by releasing them from the tube through opening the lid [20,43–50]. Adult Norway rats freed their familiar cagemates and unfamiliar conspecifics of the same strain as their cagemates but not those of an unfamiliar strain, which suggests (i) an importance of familiarity for helpful behaviour and (ii) a generalization of help directed to the phenotype of known social partners for helpful behaviour [20,44]. Ingroup bias for helping one's own familiar group members develops with age in Norway rats [46]. However, the effect of familiarity (familiar versus unfamiliar) on the help given by focal rats according to the direct reciprocity decision rules is currently unknown. The reciprocal exchange of goods and services in natural rat populations could occur between social partners by allogrooming, and by coordinated or collaborative cooperation to access food. Norway rats have been shown to reciprocally allogroom [24,41]. Natural populations of rats may adjust the returned help according to the quality of help previously received [25] and to their partner's need for help [2,18], which have both been shown in experimental studies in the laboratory. Cooperation in natural rat populations could be driven by reciprocal altruism according to the direct, generalized and indirect reciprocity decision rules [22,23] and/or by kin selection [51].

We hypothesized that focal rats should discriminate better, i.e. donate food preferentially, to previous cooperators providing more valuable help, if this help was received when food-deprived rather than when satiated, because donating food raises the chances to get something back in return [22,40]. Hence the value of received support should be appraised and increase with the scale of need. The perception of service value may depend either on the quantity or quality of the delivered commodity. Hence, when food is donated among partners, the value of this offering may be assessed by its caloric content (a measure of quantity; ‘food quantity hypothesis’) or its nutritional virtue (reflecting a quality attribute; ‘food quality hypothesis’). To determine which criterion receivers of help consider, we (i) experimentally varied the hunger status of receivers of help and (ii) donors provided either less preferred food of a higher caloric content (enhanced quantity) or preferred food higher in protein and fat content (enhanced quality) to determine which criterion rats take into account depending on their state of need. In addition, help given by focal rats in the test phase may depend on partner familiarity and previous experience with familiar partners. If focal rats integrate the outcomes from multiple previous experiences with specific partners (e.g. partners varying in their cooperation propensity versus partners being consistently cooperative), focal rats should give less help and later to partners that were unreliably cooperative during past experience than to partners that were only cooperative. After varying all these parameters in the experience phase of our experiment, the subsequent reciprocation of the received help by focal individuals in the test phase was our criterion for the rats' value assessment.

## 2. Methods

Thirty-five wild-type Norway rats were trained to pull a stick attached to a movable tray to provide food to a partner following [1,7,25]. Details about the subjects and holding conditions were given in [19,52]. The mean mass of rats was 341.5 ± 5.5 g, and the mean age of rats was 663.5 ± 0.8 d. The sample was inhomogeneous because some of the rats had met each other before our study, but information about this was lacking when we performed the experiment. There were 20 focal rats and 15 partners, and each focal rat had 3 or 4 partners. The 44 unfamiliar dyads (food-deprived and cheese: 12 dyads; food-deprived and carrots: 11 dyads; satiated and cheese: 9 dyads; satiated and carrots: 12 dyads) and the 34 familiar dyads (food-deprived and cheese: 7 dyads; food-deprived and carrots: 8 dyads; satiated and cheese: 11 dyads; satiated and carrots: 8 dyads) were combined into 78 dyads (food-deprived and cheese: 19 dyads; food-deprived and carrots: 19 dyads; satiated and cheese: 20 dyads; satiated and carrots: 20 dyads). Familiar partners had been uncooperative in an earlier experiment that had occurred 42–64 days prior. Due to the inhomogeneous nature of the sample, we report the results of the unfamiliar dyads (which corresponds to the experimental design of all previous studies on reciprocal cooperation of rats [5,7,9,25,38,53]) and the results of the familiar dyads separately. But to assess the potential familiarity effects, we first report the results of the combination of both the familiar and unfamiliar dyads.

A pilot study was performed to assess the focal rats’ preferred food by the experimenter offering a piece of cheese and a piece of carrot simultaneously (2 trials/focal rat). We recorded whether both food items were entirely eaten, and the order in which they were gathered. The pilot study evaluated the rats' preference for cheese or carrot by analysing which was eaten first with a binomial test.

**Figure 1.**
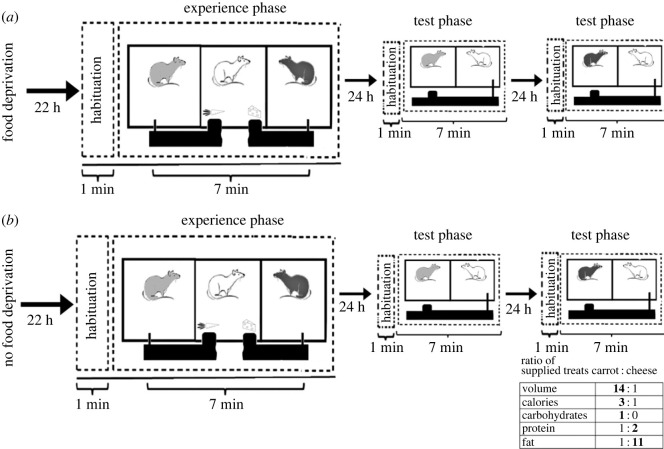
Experimental setup. Following a 22 h period, a food-deprived (*a*) or satiated (*b)* focal rat was placed in the middle compartment of a cage with two partners in the outer compartments partitioned with opaque plastic walls. In the experience phase, the focal rat received treats (nutritional ratios in the enclosed table) from both partners after a 1 min habituation. The focal rat could give help in a test phase (i) 24 h later to one of its partners and (ii) 48 h later to the other partner by pulling a stick in a two-compartment cage; partner sequence was randomized. Dr Valentina Balzarini drew the sketches of the rats.

The hunger state treatment had two levels: (i) satiated focal rats with unrestricted access to food (pellets) prior to the experience phase and (ii) food-deprived focal rats without access to food for 22 h (12 h during the resting daylight period, and 10 h during the active night-time period) prior to the experience phase (figure 1). Access to water was always provided. Experimental food items were big amounts of carrot representing a large quantity (i.e. higher calories) and low quality (i.e. lower protein and fat content and less preferred) food, and small amounts of cheese as low quantity (i.e. lower calories) and high quality (i.e. higher protein and fat content and more preferred) food (table 1). Prior to the experience phase, both partners were rubbed with paper towels, which were placed outside of the cage's compartment dividers so that the focal rat could detect the scent of its partners more easily [4]. After the random assignment of partners to the outer compartments of the experience cage, the focal rat entered the middle compartment and was given a 1 min habituation period before partners were each given a stick connected to a food tray to repeatedly pull and donate food to the focal rat for 7 min [1] (figure 1). For each pull by each partner in the experience phase, the focal rats received 1 piece of food (small cheese or large carrot). One partner donated carrots and the other donated cheese. After the experience phase, rats were returned to their home cages with free access to food and water. During each test phase (figure 1), the focal rat and one partner from the experience phase were randomly assigned to a compartment of a two-compartment cage. Following a 1 min habituation period, the focal rat was given a stick connected to the food tray to give one oat flake to its partner per pull during 7 min (i) 24 h and (ii) 48 h after the experience phase. The sequence of the presented partners was randomized.

**Table 1.** Approximate nutritional content per 100 g of the cheese and carrot [54] used in this study. Each food item was grated and trimmed to matching shapes. Cheese was cut into smaller pieces (2.86 ± 0.50 mm length) than the carrots (40.00 ± 0.50 mm length, target ratio 1 : 14) and adjusted to an estimated two-third of the calories of carrot servings, while providing twice the protein content and 11 times the fat content. The cheese's carbohydrates were negligible.

nutritional content	cheese	carrot
energy	1640 kJ (392 kcal)	172 kJ (41 kcal)
fat	32 g	0.2 g
carbohydrates	0 g	9.6 g
protein	27 g	0.9 g

Statistical analyses were performed using R [55] and the ‘lme4’ [56], ‘lmerTest’ [57], ‘survival’ [58,59], ‘frailtypack’ [60,61], ‘effects’ [62,63], ‘multicomp’ [64] and ‘ggplot2’ [65] packages. We use the top-down model selection approach [66,67]. To assess if the number of food donations by partners in the experience phase differed, we ran a generalized linear mixed model with a Poisson distribution to assess the number of pulls by partners in the experience phase with type of food (cheese versus carrots) donated in the experience phase as the fixed effect and with focal rat and partner identities as random intercept effects. We ran a generalized linear mixed model with a Poisson distribution to assess the number of pulls by focal rats in the test phase with the three-way interaction between the food type (cheese versus carrot) received in the experience phase, the hunger state (food-deprived versus satiated) of focal rats in the experience phase and familiarity (familiar versus unfamiliar partners), test sequence, time delay between the experience and test phases, and the cage side of the focal rat in the test phase (right versus left) as fixed effects. Focal rat identity was included as a random intercept effect. To assess the main effect of familiarity on the difference in help given by focal rats to familiar and unfamiliar partners, we ran the same generalized linear mixed model as the previous one, however, familiarity (familiar versus unfamiliar partners) was included as a main effect (i.e. it was not included in the interaction), and we test for a two-way interaction between the food type (cheese versus carrot) received in the experience phase and the hunger state (food-deprived versus satiated) of focal rats in the experience phase. We ran a generalized linear mixed model with a Poisson distribution to assess the number of pulls by focal rats for unfamiliar partners (i.e. unfamiliar dyads only) with the two-way interaction between type of food received in the experience phase and the hunger state of focal rats in the experience phase, test sequence, time delay between experience and test phases, and cage side as fixed effects, and with focal rat identity as a random intercept effect. The interaction between food type and hunger state was not significant, and the model represents the main effects of food type and hunger state. We ran a generalized linear mixed model with a Poisson distribution to assess the number of pulls by focal rats for familiar partner (i.e. familiar dyads only) with the interaction between type of food received in the experience phase and the hunger state of focal rats in the experience phase, test sequence, time delay between experience and test phases and cage side as fixed effects, and with focal rat identity as a random intercept effect. The residuals were not overdispersed. Interactions, test sequence, time delay and cage side were removed when non-significant.

We ran a parametric event history analysis with a Weibull distribution with the latencies to the first pulls as the response variable with a three-way interaction between the food type received in the experience phase, the hunger state in the experience phase and familiarity, test sequence, time delay between the experience and test phases, and the cage side of the focal rat in the test phase (right versus left) as fixed effects. Focal rat identity was as a shared gamma frailty. We ran two parametric event history analyses (one model with unfamiliar dyads and one model with familiar dyads) with a Weibull distribution with the latency to the first pull as the response variable with an interaction between food type received and hunger state in the experience phase, test sequence, time delay between experience and test phases and cage side as fixed effects and with focal rat identity as a shared gamma frailty. The shared frailty is similar to a random intercept effect in a generalized linear mixed model; however, the random effect has a gamma distribution. To further understand the interaction effect of food type and hunger state on the latency to the first pull by focal rats for unfamiliar partners, we ran a semi-parametric Cox proportional model with the food type and the hunger state combined into one categorical variable with four levels with focal rat identity as a random intercept effect. The proportional hazard assumption was met. Interactions, test sequence, time delay and cage side were removed when non-significant. The model results for the analyses for which the focal rats donated food to the partners in the test phase were reported without the random intercept effects for partner identity, since it did not explain any of the variance.

## 3. Results

**Figure 2.**
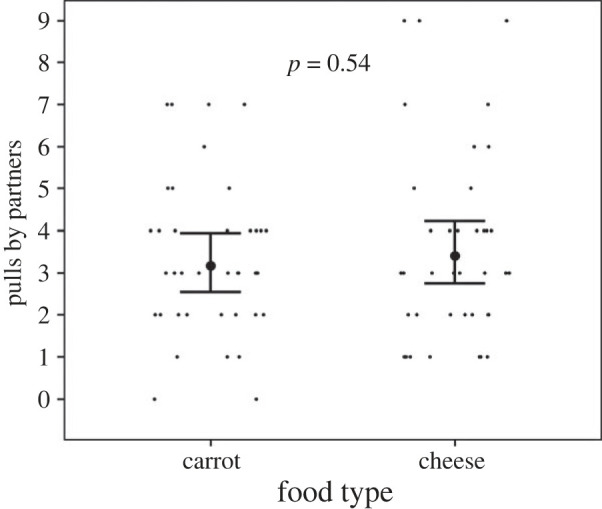
The number of pulls by partners donated to focal rats by food type in the experience phase. The large dots with the bars represent the predicted number of pulls by partners with 95% confidence intervals. The raw data are represented by the small black dots.

In the pilot study, all focal rats ate the offered cheese before eating the carrot while leaving neither behind, which was interpreted as a clear preference for cheese (binomial probability = 1.9 × 10^−6^, *p* < 0.001). In the experience phase, we detected no difference in the number of cheese and carrot donations by the partners to focal rats (Estimate ± s.e.: 0.07 ± 0.12, *p* = 0.54, figure 2, electronic supplementary material, table S1), and focal rats ate all food items donated.

### 3.1. Familiar and unfamiliar dyads combined

**Figure 3.**
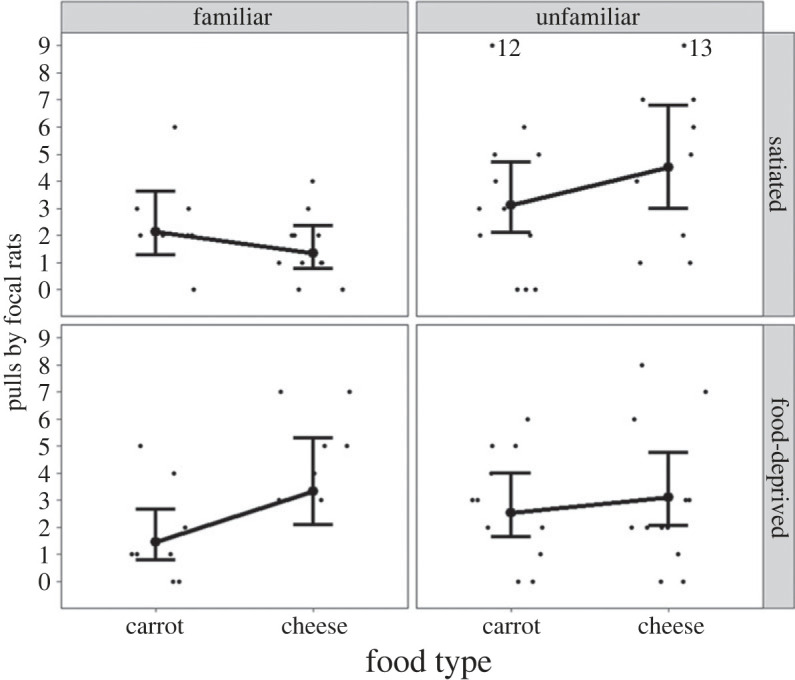
The number of pulls by focal rats in the test phase and the interaction between food type (cheese versus carrots), hunger state (food-deprived versus satiated) and familiarity (familiar versus unfamiliar). The large dots with the bars represent the predicted number of pulls by focal rats with 95% confidence intervals. The raw data are represented by the small black dots. Points labelled ‘12’ and ‘13’ represent values of 12 and 13 pulls by focal rats that are graphed here at 9 pulls, where the ordinate was truncated to enhance resolution.

**Figure 4.**
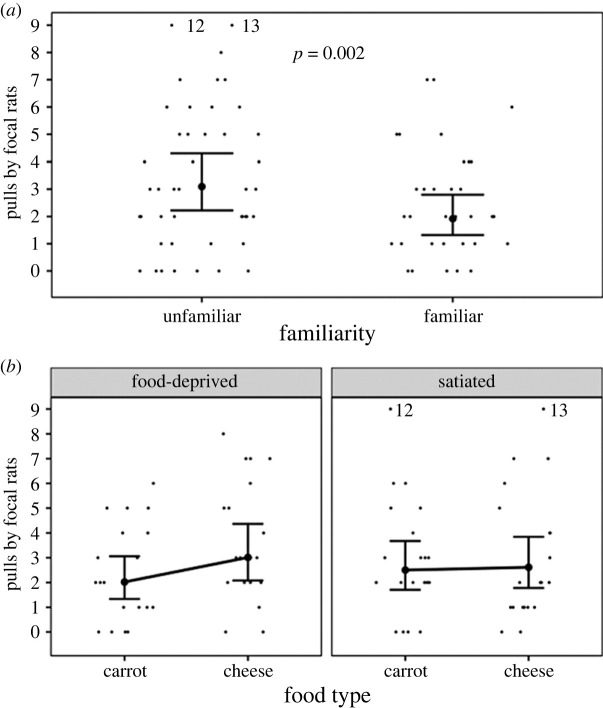
The number of pulls by focal rats in the test phase (*a*) for familiar and unfamiliar partners, and (*b*) for the interaction between food type (cheese versus carrots) and hunger state (food-deprived versus satiated). (*a*) represents the main effects of familiarity. The large dots with the bars represent the predicted number of pulls by focal rats with 95% confidence intervals. The raw data are represented by the small black dots. Points labelled ‘12’ and ‘13’ represent 12 and 13 pulls by focal rats that are graphed at 9 pulls, where the ordinate was truncated to enhance resolution.

The three-way interaction between food type, hunger state and familiarity influenced the number of pulls by focal rats in the test phase (Estimate ± s.e.: 1.45 ± 0.58, *p* = 0.01, figure 3, electronic supplementary material, table S2). We also tested the main effect of familiarity in a separate model, and the number of pulls by focal rats in the test phase was lower for familiar partners than for unfamiliar partners (main effect of familiarity (familiar versus unfamiliar): Estimate ± s.e.: −0.53 ± 0.15, *p* < 0.001, figure 4*a*, electronic supplementary material, table S3). Without familiarity in the interaction term, the number of pulls by focal rats in the test phase was not significantly influenced by the two-way interaction between food type and hunger state (Estimate ± s.e.: 0.39 ± 0.26, *p* = 0.13, figure 4*b*, electronic supplementary material, table S3). The number of pulls by focal rats in the test phase was smaller when focal rats were on the right side of the cage than on the left side of the cage (Estimate ± s.e.: −0.36 ± 0.14, *p* = 0.01, electronic supplementary material, table S3), revealing a slight side effect.

The latency to the first pull by focal rats in the test phase was not influenced by the food type received (Estimate ± s.e.: 0.31 ± 0.27 *p* = 0.24, HR (95% CI): 1.37 (0.81–2.31), electronic supplementary material, table S4) and the hunger state (Estimate ± s.e.: −0.12 ± 0.28, *p* = 0.67, HR (95% CI): 0.89 (0.51–1.54), electronic supplementary material, table S4) in the experience phase. The latency to the first pull by focal rats in the test phase was marginally longer for previously familiar partners than for previously unfamiliar partners (Estimate ± s.e.: −0.58 ± 0.30, *p* = 0.056, HR (95% CI): 0.56 (0.31–1.01), electronic supplementary material, table S4). The latency to the first pull by focal rats in the test phase was longer when focal rats were on the right side of the cage than on the left side of the cage (Estimate ± s.e.: −0.62 ± 0.29 *p* = 0.03, HR (95% CI): 0.54 (0.31–0.95), electronic supplementary material, table S4), revealing a slight side effect.

### 3.2. Unfamiliar dyads

**Figure 5.**
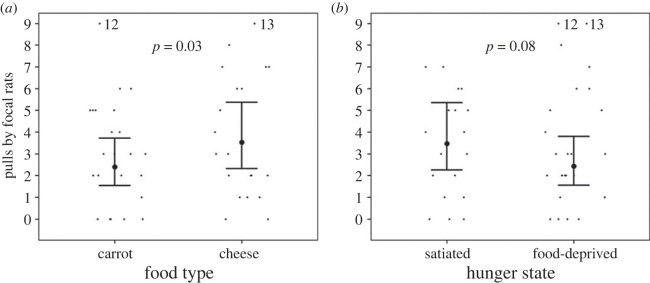
The number of pulls by focal rats for unfamiliar partners in the test phase (*a*) by food type received in the experience phase, and (*b*) by the focal rats' hunger state in the experience phase. (*a*,*b*) represent the main effects for the model without the non-significant interaction between food type and hunger state. The large dots with the bars represent the predicted number of pulls by focal rats with 95% confidence intervals. The raw data are represented by the small black dots. Points labelled ‘12’ and ‘13’ represent 12 and 13 pulls by focal rats that are graphed at 9 pulls, where the ordinate was truncated to enhance resolution.

As predicted by the food quality hypothesis, focal rats pulled more often in the test phase for partners that had donated cheese than for partners that had donated carrots in the experience phase (Estimate ± s.e.: 0.39 ± 0.18, *p* = 0.03, electronic supplementary material, table S5, figure 5*a* represents the main effect of food type without the interaction between food type and hunger state in the model), which suggests that the quality of received food is more important than the quantity of calories when deciding to return received help. Against prediction, hunger state in the experience phase did not influence the number of pulls in the test phase; if anything, there was a non-significant, opposite trend that focal rats pulled less often for their partners when they had been food-deprived in the experience phase than when they had been satiated (Estimate ± s.e.: −0.36 ± 0.20, *p* = 0.08, electronic supplementary material, table S5, figure 5*b* represents the main effect of hunger state without the interaction between food type and hunger state included in the model). The number of pulls by focal rats in the test phase was not influenced by the interaction between food type and hunger state (Estimate ± s.e.: −0.17 ± 0.34, *p* = 0.62, electronic supplementary material, table S6 represents the effect of the non-significant interaction between food type and hunger state when the interaction is included in the model with the conditional main effects).

The latency to the first pull by focal rats in the test phase was influenced by the interaction between food type and hunger state (Estimate ± s.e.: −1.84 ± 0.81, *p* = 0.02, HR (95% CI): 0.16 (0.03–0.78), electronic supplementary material, table S7). The latency to the first pull by focal rats in the test phase was longer when focal rats were food-deprived in the experience phase and pulled for partners that previously donated cheese than when focal rats were satiated in the experience phase and pulled for partners that previously donated cheese (food-deprived and cheese versus satiated and cheese: Estimate ± s.e.: −1.30 ± 0.58, *p* = 0.02, HR (95% CI): 0.27 (0.09–0.84), electronic supplementary material, table S8). The latency to the first pull by focal rats in the test phase was longer when focal rats were satiated in the experience phase and pulled for partners that previously donated carrots than when focal rats were satiated in the experience phase and pulled for partners that previously donated cheese (satiated and carrots versus satiated and cheese: Estimate ± s.e.: −1.35 ± 0.58, *p* = 0.02, HR (95% CI): 0.26 (0.08–0.80), electronic supplementary material, table S8). The latency to the first pull by focal rats in the test phase tended to be longer when focal rats were food-deprived in the experience phase and pulled for partners that previously donated carrots than when focal rats were satiated in the experience phase and pulled for partners that previously donated cheese (food-deprived and carrots versus satiated and cheese: Estimate ± s.e.: −1.11 ± 0.62, *p* = 0.07, HR (95% CI): 0.33 (0.10–1.11), electronic supplementary material, table S8). To summarize the interaction effects, focal rats in the experience phase took longer to help their partners in the test phase when (i) food-deprived and receiving cheese or carrots and (ii) satiated and receiving carrots in the experience phase than when they were satiated and receiving cheese in the experience phase. These results suggest that the value of received help increases with quality of food received (high nutritional quality, i.e. cheese, over high nutritional quantity, i.e. carrots) in a dependent way that is opposite to the scale of need (satiated, i.e. less need, greater than food-deprived, i.e. more need).

### 3.3. Familiar dyads

**Figure 6.**
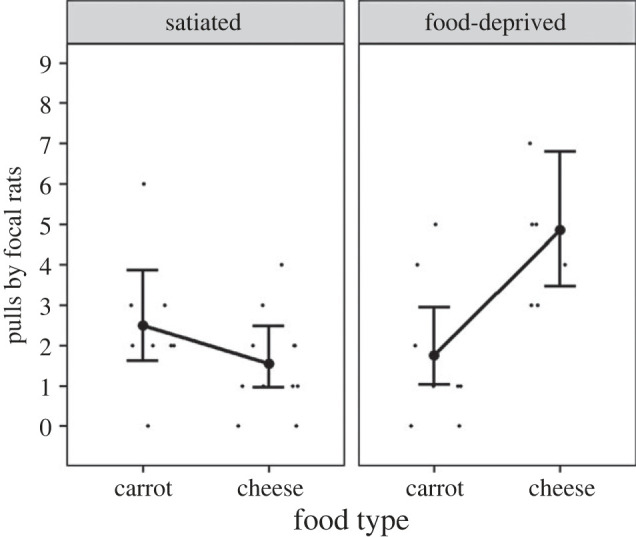
The number of pulls by focal rats for familiar partners with the interaction between food type received and hunger state. The large dots with the bars represent the predicted number of pulls by focal rats with 95% confidence intervals. The raw data are represented by the small black dots.

The interaction between food type and hunger state influenced the number of pulls by focal rats in the test phase (Estimate ± s.e.: 1.50 ± 0.46, *p* = 0.001, figure 6, electronic supplementary material, table S9). Specifically, focal rats that were food-deprived and received cheese from their partner in the experience phase pulled more often in the test phase than (i) focal rats that were food-deprived and received carrots, (ii) focal rats that were satiated and received cheese and (iii) focal rats that were satiated and received carrots in the experience phase. These results suggest that the value of received help increases in a dependent way with the scale of need and the quality of food received.

The latency to the first pull by focal rats in the test phase was not influenced by the food type received (Estimate ± s.e.: 0.18 ± 0.38, *p* = 0.64, HR (95% CI): 1.20 (0.56–2.54), electronic supplementary material, table S10) and the hunger state (Estimate ± s.e.: 0.29 ± 0.39, *p* = 0.45, HR (95% CI): 1.34 (0.63–2.85), electronic supplementary material, table S10) in the experience phase (see electronic supplementary material, S3 for additional results).

## 4. Discussion

A past study reported that focal rats gave more help to partners donating preferred food (banana) than to partners donating less preferred food (carrot); however, it was uncertain if the difference was based on the quality of food received [25]. We experimentally manipulated the quality and quantity of received help and the hunger level of focal rats as well as the familiarity status with experimental partners in the experience phase to assess the focal rats' value assessment of received help. Help given by focal rats in the test phase was the criterion for their value assessment. There was a three-way interaction between familiarity, food type and hunger state on the number of pulls by focal rats in the test phase. Focal rats pulled less often for familiar partners that were uncooperative in a previous study but cooperative in this study than for unfamiliar partners that were unanimously cooperative, and the latency to the first pull by focal rats happened marginally later for familiar partners than for unfamiliar partners. Focal rats pulled more often for unfamiliar partners from whom they received preferred food high in protein and fat (enhanced quality) than for unfamiliar partners from whom they received a less preferred food higher in carbohydrates (enhanced calories). This supports the food quality hypothesis. Focal rats that were food-deprived and received cheese from their familiar partners in the experience phase pulled more often for them. While with unfamiliar partners the focal rats’ value assessment of received help was based on the quality of food received, with previously uncooperative familiar partners, focal rats' value assessment of received help was based on the interaction between quality of food received, and the level of need.

In humans, hunger can both increase [68] and decrease [69,70] cooperation, and it was shown to increase cooperative propensity in other animals [2,18,40,52,71]. Our results suggest that satiated focal rats receiving cheese from unfamiliar partners in the experience phase accelerated their propensity to return the service in the test phase, which was in the opposite direction of our expectation based on enhanced need. However, the quality and quantity of food received and the need for help received did not affect the latency to the first pull by focal rats when they had a previously uncooperative experience with familiar partners.

The results of the three-way interaction between food type, hunger state and familiarity on the number of food donations given by focal rats in the test phase suggest that, in general, focal rats receiving cheese donations tended to increase their propensity to provide food to partners more than when they had received carrots in the experience phase, except when focal rats were satiated and receiving help from previously uncooperative, i.e. familiar, partners in the experience phase. We propose that (i) the satiated status of focal rats might cause them to respond to the previous experience with an uncooperative partner and (ii) the received food type might enhance cooperativeness except in this specific situation, i.e. receiving help from a previously uncooperative partner when being satiated in the experience phase (left upper panel of figure 3). This study was not experimentally designed to assess if the hunger status of focal rats causes them to respond to the previous experience with uncooperative partners, nor if the received food type enhances cooperativeness except when receiving help from a previously uncooperative partner while being satiated in the experience phase. Future research should assess these possibilities.

The familiarity results suggest a detrimental effect of previous uncooperative experience with the experimental partner. The main effect of familiarity on help given by focal rats in the test phase revealed that focal rats gave less help to familiar partners that had previously been uncooperative than to unfamiliar partners that were unanimously cooperative. The latency to the first pull by focal rats in the test phase was marginally longer for previously familiar partners than for previously unfamiliar partners, which further supports the detrimental effect of previous uncooperative experience with a familiar partner. The current study's familiarity results suggest that focal rats may decide to help partners according to the direct reciprocity decision rule based on the integration of the outcome of the most recent encounter with the same partner (i.e. the interaction in the experience phase of our experiment) and the outcome from a previous experience with a partner, i.e. familiarity due to previous uncooperative experience 42–62 days prior to the start of this study. It had been shown that direct reciprocity in female Norway rats is mainly based on the outcome of the most recent encounter with a specific partner, as revealed in a series of experience phases with different partners [6]. Norway rats were shown to meet the required cognitive demands of direct reciprocity over a time delay between help received and help given of up to at least 4 days [6,9]. As our data indicate that the memory of past interactions might persist much longer, future research should assess if help given by focal rats according to the direct reciprocity decision rule (i) is the same across long time delays, e.g. greater than one month, between help received and help given and (ii) is affected by an integration of the outcome from a previous experience with a partner, even if this was a long time ago, and the outcome of the most recent encounter with the same partner.

Norway rats prefer fatty foods, even when the fat itself has little nutritional value [72]. This may explain why focal rats in our study responded more strongly to the receipt of cheese, reflecting the higher quality food, than the greater caloric quantity provided by carrots. Nevertheless, the preference for cheese over carrot could have been due also to other factors (e.g. the higher protein content of cheese or the greater rarity of cheese in the standard diet; carrots were more commonly given as food than cheese in the normal diet). Because of the previous experience of the rats with both types of food it seems unlikely that focal rats had to learn the consequences of different food qualities during the experiment. We assume that the assessment of alternative food value is based on an evolved judgement via taste and/or smell perception. In a choice situation like that provided in our experiment this may cause them to prefer the food with more fat and protein (cheese) over the food available in greater quantity and containing more calories (carrots). Since animals may adjust their cooperative propensity based on expected returns [73], it would be a worthwhile goal for future studies to investigate if, given the possibility, animals preferentially choose to donate high quality food to influence their recipient's perception of their quality as a partner, and if this choice is influenced by the partner's hunger. The quantity of help provided by rats was previously shown to increase when the recipient of food donations was food-deprived [2,18,40].

The results for the latency to the first pull can differ from the number of pulls. The decision of focal rats to give help earlier to a partner may be affected by different variables than the decision of focal rats to give more help to a partner. A focal rat can pull earlier for a partner in the test phase yet pull less often in total than a different focal rat who pulls later for a partner, yet pulls more often. These are two different measures of help provided by focal rats in the test phase. Differences in results between the number of pulls and the latency to the first pull are common. For example, Dolivo & Taborsky [25] found that focal rats give help earlier to partners from which they had received bananas than from partners from whom they had received carrots in the experience phase, whereas Rutte & Taborsky [38] found that the number of pulls by focal rats was significantly influenced by the treatment but latency to the first pull by focal rats was not.

Norway rats represent a unique model system to study the behavioural mechanisms underlying reciprocal cooperation [74–76]. Previous studies showed that wild-type Norway rats return more help to partners that had provided more benefit to them before [25], and to partners that were food-deprived or in poor body condition [2,18], which can lead to enhanced begging for help [19,40,52]. The criteria rats use to appraise the received help's value to reciprocate were hitherto unclear. Hence, in this study we manipulated the quality and quantity of received help and the hunger status of rats while receiving help, as well as their familiarity with experimental partners. Our findings suggest that Norway rats assess the value of received help based on the quality rather than the quantity of food received from a partner, on partner familiarity, and on their own hunger state when receiving help. Due to a lack of similar data from other species, it is currently impossible to judge the generality of these assessment rules, but our study might prompt similar research on other systems among the great variety of species cooperating reciprocally [22,23].

## Data Availability

There is electronic supplementary material for the model results [77]. We have uploaded the Rscript and data files (.RData) to the electronic supplementary material. The Rscript can be opened in R or Rstudio. All the data files are .RData files, which can be opened in R or Rstudio.
